# Exploring DNA methylation changes in promoter, intragenic, and intergenic regions as early and late events in breast cancer formation

**DOI:** 10.1186/s12885-015-1777-9

**Published:** 2015-10-29

**Authors:** Garth H. Rauscher, Jacob K. Kresovich, Matthew Poulin, Liying Yan, Virgilia Macias, Abeer M. Mahmoud, Umaima Al-Alem, Andre Kajdacsy-Balla, Elizabeth L. Wiley, Debra Tonetti, Melanie Ehrlich

**Affiliations:** Division of Epidemiology and Biostatistics, University of Illinois-Chicago, School of Public Health, M/C 923, Chicago, IL 60612 USA; EpigenDx, Inc., Hopkinton, MA USA; Department of Pathology, University of Illinois-Chicago, Chicago, IL USA; Department of Biopharmaceutical Sciences, University of Illinois-Chicago, Chicago, IL USA; Human Genetics Program, Tulane Cancer Center, and Center for Bioinformatics and Genomics, Tulane University Health Sciences Center, 1430 Tulane Ave., New Orleans, LA 70112 USA

**Keywords:** Breast cancer, DNA methylation, Hypomethylation, Hypermethylation, Pyrosequencing, Tumor suppressor genes, Field effect, TCGA database, Transcriptome, Histone modifications

## Abstract

**Background:**

Breast cancer formation is associated with frequent changes in DNA methylation but the extent of very early alterations in DNA methylation and the biological significance of cancer-associated epigenetic changes need further elucidation.

**Methods:**

Pyrosequencing was done on bisulfite-treated DNA from formalin-fixed, paraffin-embedded sections containing invasive tumor and paired samples of histologically normal tissue adjacent to the cancers as well as control reduction mammoplasty samples from unaffected women. The DNA regions studied were promoters (*BRCA1, CD44*, *ESR1*, *GSTM2*, *GSTP1*, *MAGEA1, MSI1, NFE2L3, RASSF1A*, *RUNX3, SIX3* and *TFF1*), far-upstream regions (*EN1, PAX3, PITX2,* and *SGK1*), introns (*APC*, *EGFR*, *LHX2, RFX1* and *SOX9*) and the LINE-1 and satellite 2 DNA repeats. These choices were based upon previous literature or publicly available DNA methylome profiles. The percent methylation was averaged across neighboring CpG sites.

**Results:**

Most of the assayed gene regions displayed hypermethylation in cancer vs. adjacent tissue but the *TFF1* and *MAGEA1* regions were significantly hypomethylated (p ≤0.001). Importantly, six of the 16 regions examined in a large collection of patients (105 – 129) and in 15-18 reduction mammoplasty samples were already aberrantly methylated in adjacent, histologically normal tissue vs. non-cancerous mammoplasty samples (p ≤0.01). In addition, examination of transcriptome and DNA methylation databases indicated that methylation at three non-promoter regions (far-upstream *EN1* and *PITX2* and intronic *LHX2*) was associated with higher gene expression, unlike the inverse associations between cancer DNA hypermethylation and cancer-altered gene expression usually reported. These three non-promoter regions also exhibited normal tissue-specific hypermethylation positively associated with differentiation-related gene expression (in muscle progenitor cells vs. many other types of normal cells). The importance of considering the exact DNA region analyzed and the gene structure was further illustrated by bioinformatic analysis of an alternative promoter/intron gene region for *APC.*

**Conclusions:**

We confirmed the frequent DNA methylation changes in invasive breast cancer at a variety of genome locations and found evidence for an extensive field effect in breast cancer. In addition, we illustrate the power of combining publicly available whole-genome databases with a candidate gene approach to study cancer epigenetics.

**Electronic supplementary material:**

The online version of this article (doi:10.1186/s12885-015-1777-9) contains supplementary material, which is available to authorized users.

## Background

Aberrant DNA methylation is a hallmark of cancer [1] and may function in various ways to influence transcription, as is the case in normal differentiation [2]. Comparisons of DNA methylation in cancers to methylation in an analogous normal tissue or to methylation in a variety of normal tissues revealed that cancer is very often associated with a global reduction in DNA methylation [3–5]. Hypermethylation of promoter regions overlapping CpG islands (CpG-rich DNA sequences), most notably in some tumor suppressor genes, is also a nearly universal feature of human cancer [6–9].

Because the terms ‘hypermethylation’ and ‘hypomethylation’ indicate changes relative to some appropriate standard [10], the choice of normal tissue for comparison is critical. In cancer patients, otherwise normal-appearing tissue that is adjacent to the tumor is often used as the normal control. However, such tissue can contain early changes in DNA methylation that may contribute to tumor initiation or may just be markers of the onset of neoplasia [11, 12]. In the present study, we address the question of the prevalence of early DNA methylation changes and field effects (genetic or epigenetic abnormalities in tissues that appear histologically normal) in breast cancer development using paired adjacent normal and invasive tissue from a total of 129 patients with breast cancer together with 18 reduction mammoplasty controls from cancer-free women. The DNA regions examined for differential methylation included promoters, far-upstream regions, and introns as well as DNA repeats. The gene-associated regions included tumor suppressor genes, stem cell-associated genes and transcription factor genes. The regions for analysis were chosen using findings from the literature and bioinformatics, especially epigenetic data from the Encyclopedia of DNA Elements (ENCODE) at the UCSC Genome Browser [13]. We also used bioinformatics to compare our DNA methylation results with those in The Cancer Genome Atlas (TCGA) [14], one of the most comprehensive public databases on DNA methylation changes in breast cancer. To elucidate the biological significance of our findings, we examined whole-genome expression data for breast cancers from TCGA as well as DNA epigenetic, chromatin epigenetic and transcriptome profiles from cell cultures represented at the UCSC Genome Browser [13, 15]. Our results provide evidence for frequent field effects in breast cancer development and illustrate the power of combining whole-genome epigenome and transcriptome profiles with examination of individual gene regions.

## Methods

### Source of samples

Breast cancer patients (N = 129) came from the Breast Cancer Care in Chicago (BCCC) study and were diagnosed at one of many Chicago area hospitals. The study was approved by the University of Illinois at Chicago institutional review board. Women were between the ages of 30 and 79, self-identified as non-Hispanic White, non-Hispanic Black or Hispanic, resided in Chicago, had a first primary in situ or invasive breast cancer diagnosed between 2005 and 2008 and gave written consent to participate in the study and to allow the research staff to obtain samples of their breast tumors from diagnosing hospitals. In addition, 18 unaffected, cancer-free patients who underwent a reduction mammoplasty between 2005-2008 served as non-cancerous controls. The 18 control tissues were made available through a standardized protocol involving an honest broker within the UIC department of pathology. For all patients, hematoxylin and eosin (H&E) stained slides from formalin-fixed, paraffin-embedded (FFPE) tumor blocks were examined to determine representative areas of invasive tumor, histologically and morphologically normal-appearing breast tissue adjacent to the tumor, or confirmed histologically normal tissue obtained from reduction mammoplasty samples (referred to as control or ‘non-cancerous’ samples). For lumpectomies, adjacent breast tissue was usually chosen from the same block as the tumor. However, when available, a separate block containing breast tissue and no tumor was used as the non-malignant, adjacent sample. Tissue core samples were precisely cut from the selected area using a semiautomated tissue arrayer (Beecher Instruments, Inc.). Because the tissue was fixed and sealed by paraffin, cells from the invasive tissue could not become dislodged and contaminate the adjacent tissue or vice versa.

### DNA methylation analysis

Dissolution of paraffin was accomplished by the addition of 1 mL of clearing agent (Histochoice) and incubation at 65 °C for 30 min. Samples were digested by the addition of 100 μL of digestion buffer consisting of 10 μL 10X Target Retrieval Solution high pH (DAKO, Glostrup, Denmark), 75 μL of ATL Buffer (Qiagen), and 15 μL of proteinase K (Qiagen) and incubation at 65 °C overnight. They were then vortexed and checked for complete digestion. The sample volume was brought up to ~100 μL, and 20 μL of each sample was treated with bisulfite and purified using the Zymo EZ-96 DNA Methylation-Direct™ Kit, with a 15-min denaturation step at 98 °C followed by a 3.5-h conversion at 64 °C, an additional 15-min denaturation at 98 °C and a 60-min incubation at 64 °C. DNA was eluted in 40 μL of elution buffer. Then, PCR was performed with 0.2 μM of each primer, one of which was biotinylated, and the final PCR product was purified (Streptavidin Sepharose HP, Amersham Biosciences, Uppsala, Sweden), washed, alkaline-denatured, and rewashed (Pyrosequencing Vacuum Prep Tool, Qiagen). Then, pyrosequencing primer (0.5 μM) was annealed to the purified single-stranded PCR product, and 10 μL of the PCR products were sequenced by Pyrosequencing PSQ96 HS System (Biotage AB) following the manufacturer’s instructions. The amplicon regions used are given in Table 1. The methylation status of each locus was analyzed individually as a T/C SNP using Pyromark Q96 software (Qiagen, Germantown, Maryland).

**Table 1:** List of studied gene regions and number of CpGs covered, Breast Cancer Care in Chicago study (2005-2008)

Gene/RNA isoform^a^	Test region	Test region coordinates (hg19)	Distance from TSS (bp)^b^	CGI^c^	# CpGs^d^	TSG^e^
Promoter region						
BRCA1	Exon 1 (extended promoter)	chr17: 41277463-41277365	+37 to +135	No	11	Yes
CD44	Promoter	chr11: 35160374-35160443	-43 to +26	Yes	8	No
ESR1	Exon 2 (extended promoter)	chr6: 152129110 - 152129167	+656 to +713	Yes	5	Unclear
GSTM2	Promoter	chr1: 110210582-110210641	-62 to -3	Yes	8	No
GSTP1	Exon 1 (extended promoter)	chr11: 67351205-67351215	+139 to +149	Yes	4	Yes
MAGEA1^f^	Promoter	chrX: 152486180-152486129	-13 to -64	No	6	No
MSI1	Promoter	chr12: 120807571-120807474	-588 to -491	No	5	
NFE2L3	Exon 1 (Extended promoter)	chr7: 26192663-26192744	+816 to +897	Yes	14	No
RASSF1A	Exon 1 (extended promoter)	chr3: 50378293-50378233	+74 to +134	Yes	9	Yes
RUNX3	Exon 1 (extended promoter)	chr1: 25256198-25256306	+464 to +572	Yes	28	Yes
SIX3	Exon 1 (extended promoter)	chr2: 45169609-45169529	+492 to +572	Yes	12	Unclear
TFF1	Promoter	chr21: 43786664-43786628	-20 to +16	No	5	Unclear
Upstream of promoter						
EN1	Upstream of promoter	chr2: 119611385-119611338	-5579 to -5626	Yes	6	No
PAX3^g^	Far upstream	chr2: 223170608-223170643	-6928 to -6893	Yes	5	No
PITX2^f,h^	Far upstream or intron I	chr4: 111562566-111562677	-18312 to -18413/+602 to +713	No	10	No
SGK1^h^	Far upstream/ alt. exon 1	chr6: 134638893-134638831	-14823 to -14761/+303 to +365	Yes	6	Unclear
Introns						
APC	Intron 1 or promoter	chr5: 112073426-112073445	+30224 to +30243/-130 to -111	No	4	Yes
EGFR	intron 1	chr7: 55088080-55088104	+1355 to +1379	Yes	4	No
LHX2	Intron 3	chr9: 126777854-126777983	+3966 to +4095	Yes	11	No
RFX1^f^	Intron 7	chr19: 14089984-14089969	+27150 to +27165	No	4	No
SOX9	Intron 2	chr17: 70119151-70119195	+1990 to +2034	Yes	4	No
DNA Repeats						
LINE-1	N.A.	DNA Repeat	N.A.		4	N.A.
Sat2	N.A.	DNA Repeat	N.A.		2	N.A.

^a^Where there are multiple RefSeq RNA isoforms and expression in HMEC cells by RNA-seq (ENCODE/Cold Spring Harbor), the RNA isoform closest to the predominant HMEC RNA was used in this table to determine the TSS. The isoforms for calculation of the distance from the TSS are given in Additional file 1: Tables S1 and S2

^b^TSS, transcription start site for the indicated RefSeq isoform. N.A., not applicable

^c^CGI, CpG island overlapping the test region

^d^The number of CpG dinucleotide pairs in the test region (the amplicon used for pyrosequencing minus the primer regions)

^e^TSG, Tumor suppressor gene

^f^Although the sequences were in regions that did not make the criteria to be classified as CGI [13], the regions were rich in CpG compared to the average for human DNA

^g^There is a little expressed, primate specific gene, *CCDC140*, between *PAX3* and the test region whose 5’ end overlaps the 5’ end of *PAX3*

^h^There are distant alternative 5’ ends of these genes

### Quality control of DNA methylation analysis

All primer-pairs passed tests for sensitivity, reproducibility, and lack of amplification bias (EpigenDx, Hopkinton, MA). All reactions had negligible levels of persisting non-CpG cytosine residues. For each set of PCR primers, a dilution series of technical triplicates was examined with ≤15 ng bisulfite-treated DNA. Primer-pairs were discarded if the signal for a single nucleotide peak was below 50 relative light units (RLU’s). The signal to noise (S/N) ratio was calculated by dividing the RLU signal from a single nucleotide incorporation by the RLU value from a negative control nucleotide incorporation, and primer-pairs were discarded if the S/N ratio was less than 10. The reproducibility of percent methylation was also assessed and primer-pairs were excluded if the coefficient of variation exceeded 5 %. The lack of amplification bias was demonstrated for each utilized primer-pair by mixing different relative amounts of human placental DNA (Bioline, Taunton, MA) that had been methylated (with SssI-methyltransferase) and amplified DNA left unmethylated (HGHM5 and HGUM5, EpigenDx). The empirically determined methylation values were compared with the known values. An R-square value of >0.9 was required for validation.

### Statistical analysis

#### Breast Cancer Care in Chicago pyrosequencing study

We conducted pyrosequencing methylation assays on 276 FFPE samples including 258 samples of paired invasive and adjacent tissue from 129 patients with invasive breast cancer, as well as 18 reduction mammoplasty non-cancerous controls. Methylation values were averaged across multiple neighboring CpG sites to create a single value for percent methylation for each assay. Mean and 95 % confidence intervals for percent methylation were estimated for each gene separately for control mammoplasty, adjacent and cancer samples. Differences in means between unpaired control mammoplasty vs. adjacent and cancer tissues were evaluated via p-values from independent Wilcoxin rank-sum tests, whereas differences in means between paired adjacent and cancer tissues were evaluated via p-values from dependent Wilcoxon signed-rank tests. Differences in means between adjacent and cancer tissues were also estimated in linear regression with generalized estimating equations to account for the paired nature of the samples, and 95 % confidence intervals were estimated via 1000 bootstrap replications with bias correction. These models were adjusted for patient age, race/ethnicity and tumor characteristics (stage at diagnosis, tumor grade and either adjusted for or stratified by ER/PR status). For differential methylation in cancer vs. adjacent tissue at DNA regions in the complete sample set, we used a significance level of p ≤ 0.001. For those DNA regions not pursued beyond the pilot phase, which were examined in only 37 pairs of cancer and adjacent tissue, we used a significance level of p ≤ 0.01.

#### The Cancer Genome Atlas (TCGA) bioinformatics study

We examined methylation results for 192 samples of paired breast cancers and normal tissue (N = 96), based on TCGA profiles [14] from the Infinium HumanMethylation450 array performed on frozen (not formalin fixed) samples. Differences in mean methylation between paired normal and invasive tissues were evaluated using p-values from dependent Wilcoxon signed-rank tests.

Additionally, to examine the correlation between regional methylation and gene expression values, invasive breast cancer tumors with both methylation results and gene expression results (N = 800) were obtained from TCGA bioportal [16, 17]. Methylation value data were aquired using the Infinium HumanMethylation450 assay and gene expression data were taken as z-scores using Illumina HighSeq 2000 Total RNA Sequencing Version 2. Spearman correlation coefficients were calculated to measure the association between regional loci methylation level and gene expression level. The level for significance for both of the previously identified analyses was defined as p ≤ 0.01. Lastly, other whole-genome databases that are part of the ENCODE project [18, 19] and publicly available profiles for all mappable CpGs in control and cancer-derived breast epithelial cell cultures using next-generation sequencing of bisulfite-treated DNA (bisulfite-seq) [15] were examined for DNA methylation, transcription, or histone modification as described in Results.

## Results

### Choice of regions for analysis

We chose a diverse set of genes and two DNA repeats (Table 1) to assay for DNA methylation in cancer, adjacent and control mammoplasty tissues. Eight of the 23 examined DNA regions overlapped or were near regions previously reported to be hypermethylated in breast cancer vs. non-cancerous breast tissue, namely, *EGFR* [20], *GSTP1* [21], *LHX2* [22], *PITX2* [23], *RASSF1A* [24], *RUNX3* [25], *APC* [26] and *BRCA1* [27, 28] or hypomethylated in breast cancer vs. normal breast, namely, *TFF1* [29], satellite 2 and LINE-1, DNA repeats [30, 31]. In addition, the first six of the above-mentioned gene regions displayed hypermethylation in one or two breast cancer cell lines (MCF-7 and T-47D) relative to a human breast epithelial cell culture derived from normal breast tissue (human mammary epithelial cells, HMEC) and compared with most normal tissues, including breast tissue as seen in whole-genome DNA methylation data (reduced representation bisulfite sequencing, RRBS) from the ENCODE project [5, 13, 19]. An additional seven gene regions (*EN1, PAX3, SIX3, SOX9, RFX1, SGK1* and *NFE2L3*) were chosen mostly on the basis of hypermethylation profiled by RRBS in breast cancer cells lines (and often other cancer cell lines) vs. the above-mentioned normal cell cultures or tissues [13]. The first five of these genes also had been previously reported to display hypermethylation in non-breast neoplasms vs. control tissue [32–35].

**Fig. 1:**
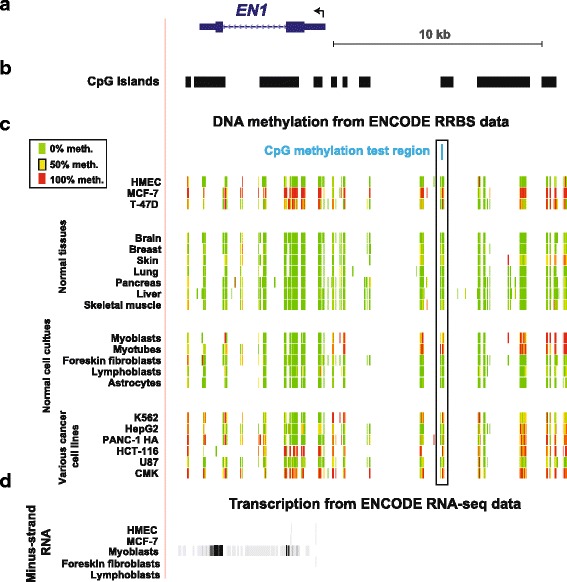
Example of how some gene regions were chosen for examination in this study on the basis of available RRBS DNA methylation profiles for breast cancer cell lines and normal cell cultures and tissues visualized in the UCSC Genome Browser [13]. **a** The *EN1* gene structure with exons as heavy horizontal bars; **b**, the aligned CpG islands in the illustrated region.; **c**, DNA methylation (ENCODE/RRBS/HudsonAlpha) profiles for the indicated cell cultures and normal tissues using an 11-color, semi-continuous scale (see color key) to indicate the average DNA methylation levels at each monitored CpG site; **d**, aligned transcription results indicating that the non-transformed breast cancer cell line is not transcribing this gene irrespective of its lack of DNA methylation. Paradoxically, normal myoblasts are transcribing it despite some upstream DNA methylation. All data are from ENCODE [19]

Figure 1 illustrates ENCODE data at the UCSC Genome Browser [13] for the studied region far upstream of *EN1*, one of the gene regions chosen for examination in this study on the basis of RRBS DNA methylation data for breast cancer cell lines vs. control cells and tissues. *EN1* encodes a homeobox-containing transcription factor that is implicated in the development of the nervous system and serves as a marker of certain neurons [36]. Underneath the diagrammed gene structure (Panel a) are the aligned CpG islands in the illustrated region (Panel b). The tracks in Panel c show the DNA methylation status quantified at the RRBS-detected CpGs in a variety of cell cultures and normal tissues using an 11-color, semi-continuous scale (see color key) to indicate the average DNA methylation levels at each monitored CpG site (ENCODE/RRBS/HudsonAlpha Institute, [13]). The MCF-7 breast cancer cell line and several diverse cancer cell lines were hypermethylated throughout most of the gene and its upstream region relative to HMEC, normal breast tissue, other normal tissues and the majority of non-cancer cell cultures (Panel c and data not shown from ENCODE [13]). The exceptions were normal muscle cell cultures (myoblasts and myotubes) but these were methylated in a smaller region that did not overlap the beginning of the gene as did the hypermethylation in MCF-7 cells. T-47D, the second examined breast cancer cell line in this RRBS database, was hypermethylated relative to HMEC but to a lesser extent than for MCF-7 cells.

We also examined two gene regions (*ESR1* and *GSTM2*) found to display hypermethylation preferentially in more aggressive breast cancers [37, 38]. In addition, we studied *CD44* and *MSI1,* which have been reported to have promoter hypomethylation in triple-negative breast cancers, that is, cancers that lack estrogen receptors (ER), progesterone receptors (PR), and human epidermal growth factor-2 receptors (HER2) [39]. The last gene region we examined was *MAGEA1,* which encodes a cancer-testis antigen that is not expressed in normal somatic tissues but is sometimes expressed in breast cancer [40]. Cancer-testis antigen genes are often hypomethylated in various kinds of cancer [41], although the methylation status of *MAGEA1* in breast cancer was not known.

### Samples and method used for DNA methylation analysis

The breast tissue samples analyzed for DNA methylation were invasive cancer (referred to as “cancer”), histologically normal tissue adjacent to the cancer (referred to as “adjacent tissue”) and non-cancerous reduction mammoplasty samples (referred to as “control mammoplasty”). Characteristics of the 129 breast cancer patients and their tumors are listed in Table 2. The carcinomas were equally likely to be stage I vs. later stages, equally distributed across histological grades, and one third of them lacked both estrogen and progesterone receptors. Before studying the full sample set, we conducted a pilot study on the 23 test regions using paired samples of cancer and adjacent tissue from 37 patients, and on samples from 18 reduction mammoplasty patients. Of the 23 test regions, 16 were analyzed in an additional set of 92 patients with paired cancer and adjacent tissue samples to give a total of 276 samples.

**Table 2:** Characteristics of the 129 breast cancer patients with adjacent normal and/or invasive samples, Breast Cancer Care in Chicago study (2005-2008)

Patient characteristic	No.	Percent
Age		
<50	44	34
50-59	36	28
60-69	32	25
70-79	17	13
Race/Ethnicity		
non-Hispanic White	42	33
non-Hispanic Black	57	44
Hispanic	30	23
Stage at Diagnosis		
Stage I	60	47
Stage II-IV	68	53
Histologic Grade		
Low	39	32
Intermediate	42	34
High	42	34
ER/PR status		
One or both positive	82	67
Double negative	40	33

Methylation analysis was performed by pyrosequencing of bisulfite-treated DNA. This method allowed us to monitor individual reactions for incomplete bisulfite modification and to check for PCR-bias [42, 43]. We used FFPE-derived DNA, which is partly degraded and difficult to analyze because of crosslinking resulting from the formalin fixation process [44], and which may be available in only small amounts. These problems are compounded by further degradation associated with bisulfite treatment for the methylation analysis. Bisulfite-based pyrosequencing overcomes these problems and provides accurate quantification [43].

### Variation in DNA methylation among samples of the same tissue type

As expected for cancer-linked DNA methylation changes [7], there was large variability in the average 5-methylcytosine (5mC) content at a given test region among individual cancer samples, as seen in the high standard deviation (SD) relative to the mean methylation values (Table 3). The between-sample variability contrasted with the much lower within-sample variability of technical duplicates (data not shown), observed in the pilot study. Moreover, the control mammoplasty samples generally showed less variability in average 5mC content compared with adjacent or cancer samples (Table 3).

**Table 3:** Mean percent methylation by gene and tissue type from the Breast Cancer Care in Chicago study

DNA region	Control^a^			Adjacent^b^			Invasive^c^			Adjacent vs. control		Invasive vs. control		Invasive vs. adjacent	
	N	Mean	SD	N	Mean	SD	N	Mean	SD	Diff.	*P*-value^d^	Diff.	*P*-value^d^	Diff.	*P*-value^e^
Promoter region															
BRCA1	15	1.6	1.1	105	1.4	4.3	105	3.0	9.6	-0.2	NS	1.4	NS	1.6	0.031
CD44^f^	18	1.6	0.6	37	1.8	0.9	37	2.6	2.7	0.2	NS	1.0	NS	0.8	NS
ESR1^f^	18	5.9	3.1	37	7.9	6.0	37	6.7	7.8	2.0	NS	0.8	NS	-1.2	NS
GSTM2	16	1.8	2.0	107	3.0	6.1	107	19.3	22.9	1.2	NS	17.5	0.004	16.3	<0.0001
GSTP1	17	2.5	2.0	118	1.6	3.7	118	9.9	16.8	-0.9	NS	7.4	NS	8.3	<0.0001
MAGEA1^f^	17	84.8	4.9	32	84.2	4.8	37	67.0	16.2	-0.6	NS	-17.8	0.0001	-17.2	<0.0001
MSI1^f^	18	3.8	2.1	36	4.2	2.5	37	9.2	7.4	0.4	NS	5.3	0.0001	5.0	0.0003
NFE2L3^f^	17	28.9	14.7	36	26.9	13.7	37	33.7	24.4	-2.0	NS	4.8	NS	6.8	NS
RASSF1A	18	2.8	2.2	124	9.7	10.7	124	33.3	23.4	6.9	<0.0001	30.5	<0.0001	23.6	<0.0001
RUNX3	17	3.3	1.2	119	4.0	3.3	119	11.4	12.7	0.7	NS	8.1	0.061	7.4	<0.0001
SIX3	17	5.8	2.8	115	5.3	4.0	115	15.7	14.9	-0.5	NS	9.9	0.026	10.4	<0.0001
TFF1	18	81.8	5.4	122	72.0	16.9	122	49.2	22.3	-9.8	0.008	-32.6	<0.0001	-22.8	<0.0001
Upstream of promoter															
EN1	18	17.8	5.3	122	20.0	10.2	122	32.9	15.3	2.2	NS	15.1	<0.0001	12.9	<0.0001
PAX3	17	2.9	1.2	121	4.0	5.2	121	10.8	11.1	1.1	NS	7.9	0.0003	6.8	<0.0001
PITX2^f^	17	26.5	6.1	35	27.6	8.6	36	36.1	11.3	1.1	NS	9.6	0.001	8.5	<0.0001
SGK1	18	1.6	1.2	124	3.9	3.7	124	13.0	12.2	2.3	<0.0001	11.4	<0.0001	9.1	<0.0001
Introns															
APC	18	2.0	1.9	114	2.9	8.3	114	14.8	19.6	0.9	NS	12.8	0.058	11.9	<0.0001
EGFR	18	4.5	1.3	126	7.3	5.2	126	19.4	14.8	2.8	0.006	14.9	<0.0001	12.1	<0.0001
LHX2^f^	18	21.1	5.3	36	25.8	13.1	37	36.1	12.6	4.7	NS	15.0	<0.0001	10.3	0.0007
RFX1	18	18.0	5.3	126	19.3	9.7	126	39.4	13.1	1.3	NS	21.4	<0.0001	20.1	<0.0001
SOX9	18	8.4	4.3	123	9.1	7.4	123	15.2	14.3	0.7	NS	6.8	NS	6.1	0.002
DNA Repeats															
LINE-1	18	68.7	1.4	129	72.8	2.6	129	71.2	4.3	4.1	<0.0001	2.5	0.0003	-1.6	0.001
Sat2	18	52.6	8.0	128	57.4	12.7	128	52.0	13.4	4.8	0.002	-0.6	NS	-5.4	<0.0001

^a^Reduction mammoplasty samples from women unaffected with breast cancer

^b^Samples from histologically normal tissue adjacent to the tumor

^c^Samples from the cancer component of the tumor

^d^From an independent sample Wilcoxon Rank Sum test comparing control mammoplasty vs. adjacent samples

^e^From a dependent sample Wilcoxon Sign Rank test. P-values > 0.10 are suppressed. Diff, difference in mean methylation; SD, standard deviation

^f^These seven assays were not pursued beyond the pilot phase and, therefore, had 32-37 paired cancer and adjacent samples instead of 105-129

Differences were determined to be statistically significant at p < 0.001 for the complete sample set and p < 0.01 for regions only examined in the pilot study

### DNA hypermethylation in cancer vs. adjacent and control mammoplasty samples

**Fig. 2:**
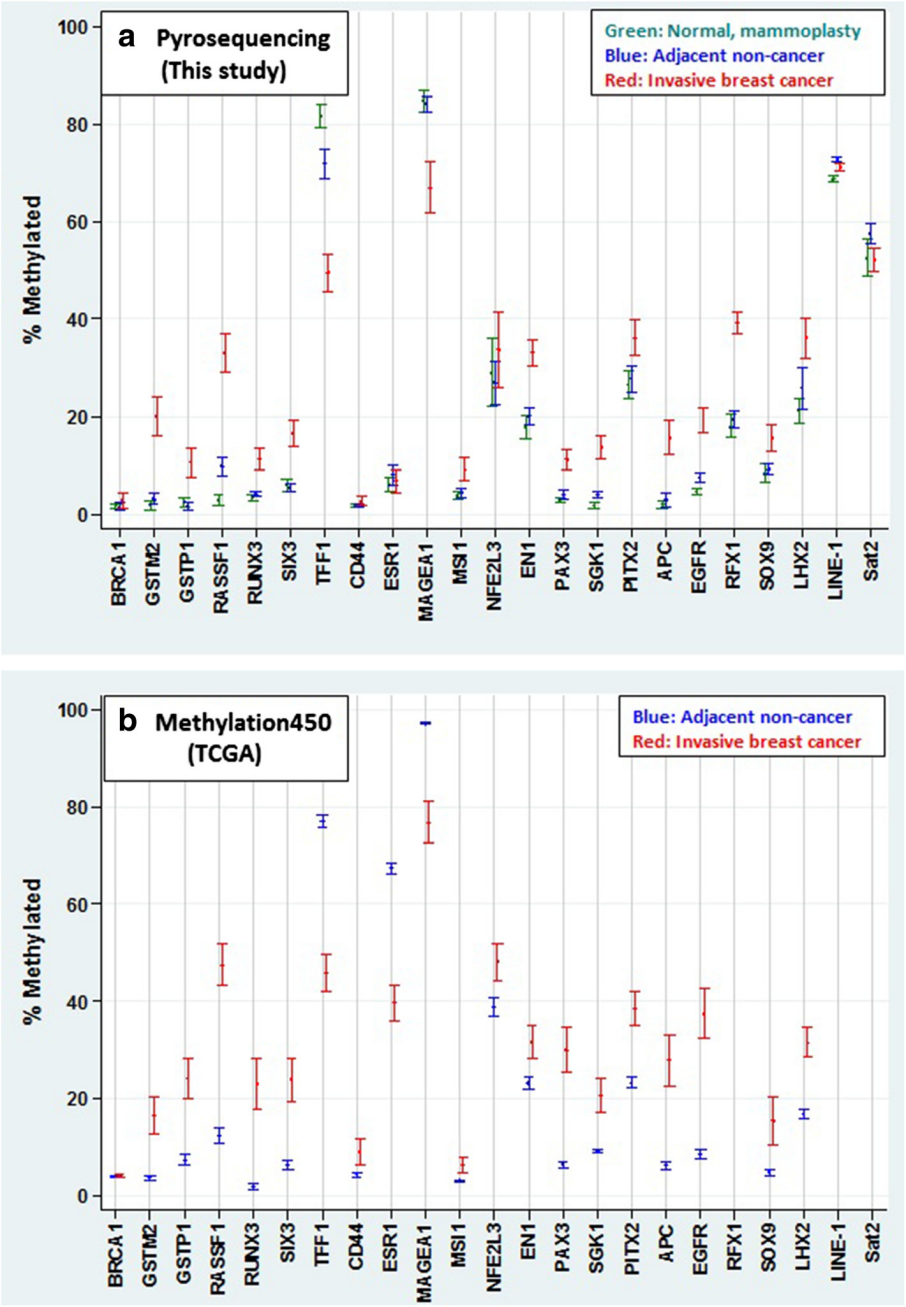
Mean percent methylation and 95 % error bars by gene and tissue type for the DNA regions listed in Table 1. **a** DNA methylation analysis of samples from the Breast Cancer Care in Chicago study (2005-2008) as determined by our bisulfite pyrosequencing. Control samples (reduction mammoplasty) from unaffected women are represented by green bars, cancer-adjacent, histologically normal samples by blue bars and cancer samples by red bars. **b** Bioinformatic analysis of DNA methylation of breast cancer samples and paired non-cancerous adjacent samples from The Cancer Genome Atlas (TCGA). Paired non-cancerous adjacent samples are represented by blue bars and cancer samples by red bars. In both panels, promoter sequences are displayed first, followed by upstream sequences, then introns and lastly, DNA repeats

Figure 2 (Panel a) displays the mean percent methylation and 95 % confidence limits for each of the 23 studied DNA regions and shows the results separately for control mammoplasty, adjacent and cancer samples. Hypermethylation in cancer vs. adjacent samples was seen at a significance level of p ≤ 0.001 for 12 of the 16 test regions in the large-scale study and at a significance level of p ≤ 0.01 for three of the seven regions not pursued beyond the pilot phase (Table 3). Twelve of the regions were also significantly hypermethylated in cancer vs. control mammoplasty samples (p ≤ 0.01) (Table 3). The difference in the average percent methylation for significiantly hypermethylated sequences in cancer vs. adjacent tissue or for cancer vs. control mammoplasty tissue was largest for *RASSF1A* (23.6 and 30.5, respectively). Cancer-associated hypermethylation was seen in test sequences that were in extended promoter regions (regions immediately upstream or downstream of the transcription start site, TSS), in sequences upstream of promoter regions and in introns. A mostly similar pattern of cancer hypermethylation of these gene regions was observed in TCGA for breast cancer and paired normal samples (Fig. 2, panel b).

Eight of the ten test regions overlapping DNA sequences previously reported to be hypermethylated in breast cancer vs. nonmalignant breast tissue or in more aggressive vs. less aggressive cancer types (*APC , EGFR, GSTM2, GSTP1, LHX2, PITX2, RASSF1A* and *RUNX3*) exhibited hypermethylation in this study at the designated p-value cutoff levels (p < 0.001 and p < 0.01, Table 3). Two other genes (*BRCA1* and *ESR1*) displayed very small changes in the extent of methylation (<2 % differential for cancer vs. adjacent tissue). *BRCA1* methylation was low for all three tissue types, ranging from a mean of 1 % in adjacent to only 3 % in cancer samples. However, *BRCA1* showed the largest relative SD of all tested regions (>3-fold, Table 3). Four percent of cancer samples and none of the adjacent or control mammoplasty samples displayed *BRCA1* methylation in excess of 20 % (results not shown). For additional DNA regions that were hypermethylated in breast cancer cell lines or in cancers other than breast (*EN1, NFE2L3, PAX3, RFX1, SGK1*, *SIX3* and *SOX9*), significant hypermethylation was seen in the cancer tissue compared with adjacent tissue with the exceptions of *SOX9* (p = 0.002) and *NFE2L3* (Table 3).

Results were not substantively different after adjusting for patient and tumor characteristics (age, race/ethnicity, ER/PR status, stage and grade) (Table 4). When stratifying estimates by ER/PR status, several genes appeared to display differential changes in methylation for adjacent vs. cancer tissues (Table 4). *GSTM2* exhibited more hypermethylation for ER/PR negative tumors (p < 0.05), whereas *EGFR* displayed greater hypermethylation for ER/PR positive tumors (p < 0.05). *TFF1* and *MAGEA1* displayed greater hypomethylation for ER/PR positive tumors. *NFE2L3* displayed hypermethylation for ER positive tumors and hypomethylation for ER negative tumors (p < 0.05) (Table 4).

**Table 4:** Adjusted differences in mean % methylation comparing adjacent (referent) to cancer tissue, overall and stratified by ER/PR status

	All samples				ER/PR Positive				ER/PR Negative			
	N^a^	Diff.^b^	95 % CI^c^	*P*-Value^d^	N^a^	Diff.^b^	95 % CI^c^	*P*-Value^d^	N^a^	Diff.^b^	95 % CI^c^	*P*-Value^d^
Promoter regions												
BRCA1	198	1.7	(0, 4)	NS	136	1	(-1, 4)	NS	62	3	(0, 8)	0.09
CD44^e^	54	0.5	(0, 1)	NS	32	0	(-0.4, 1)	NS	22	0	(0,2)	NS
ESR1^e^	54	-1.5	(-3, 0)	0.099	32	-1	(-4, 1)	NS	22	-2	(-4, 1)	NS
GSTM2	212	16.8	(13, 21)	< 0.0001	146	10	(6, 16)	< 0.0001	66	32	(24, 38)	< 0.0001
GSTP1	227	8.3	(6, 12)	< 0.0001	159	9	(6, 14)	< 0.0001	68	6	(2, 12)	0.016
MAGEA1^e^	54	-14.5	(-22, -9)	< 0.0001	32	-22	(-30, -14)	< 0.0001	22	-4	(-12, 3)	NS
MSI1^e^	54	4.7	(2, 8)	0.005	32	4	(1, 10)	0.057	22	5	(1, 9)	0.01
NFE2L3^e^	54	5.6	(-4, 16)	NS	32	19	(9, 31)	0.001	22	-15	(-26, -4)	0.01
RASSF1A	234	23.5	(19, 28)	< 0.0001	160	26	(21, 31)	< 0.0001	74	18	(12, 25)	< 0.0001
RUNX3	227	6.6	(4, 9)	< 0.0001	156	9	(6, 11)	< 0.0001	71	2	(-1, 7)	NS
SIX3	221	10.9	(9, 14)	< 0.0001	151	10	(7, 14)	< 0.0001	70	13	(8, 19)	< 0.0001
TFF1	230	-21.6	(-26, -17)	< 0.0001	159	-25	(-30, -19)	< 0.0001	71	-14	(-23, -5)	0.002
Upstream of promoter												
EN1	230	13.1	(10, 17)	< 0.0001	158	12	(8, 16)	< 0.0001	72	16	(9, 24)	< 0.0001
PAX3	230	7.3	(5, 10)	< 0.0001	159	6	(4, 9)	< 0.0001	71	10	(6, 15)	< 0.0001
PITX2^e^	54	7.8	(4, 11)	< 0.0001	32	7	(3, 10)	< 0.0001	22	10	(1, 16)	0.026
SGK1	233	9.8	(8, 12)	< 0.0001	160	9	(7, 12)	< 0.0001	73	11	(7, 16)	< 0.0001
Introns												
APC	221	12.3	(9, 16)	< 0.0001	153	12	(8, 16)	< 0.0001	68	15	(9, 22)	< 0.0001
EGFR	235	12	(9, 15)	< 0.0001	161	15	(11, 18)	< 0.0001	74	6	(2, 11)	0.009
LHX2^e^	54	8.1	(2, 15)	0.017	32	8	(3, 13)	0.006	22	9	(-5, 22)	NS
RFX1	235	19.8	(17, 23)	< 0.0001	161	20	(16, 23)	< 0.0001	74	21	(16, 26)	< 0.0001
SOX9	231	6.2	(3, 9)	< 0.0001	158	6	(3, 10)	< 0.0001	73	6	(1, 13)	0.031
DNA Repeats												
LINE-1	238	-1.5	(-2, -1)	< 0.0001	162	-2	(-3, -1)	< 0.0001	76	-1	(-3, 0)	0.074
Sat2	237	-5.4	(-9, -2)	0.001	161	-6	(-10, -2)	0.003	76	-5	(-10, 1)	0.109

^a^Number of samples analyzed; the small differences in numbers of samples in this table compared to Table 3 are due to missing data on ER/PR status

^b^Difference in mean percent methylation (cancer and adjacent) was estimated via linear regression model with generalized estimating equations to account for within-patient covariance

^c^Bias corrected, bootstrapped 95 % confidence intervals (CI) were estimated via 1000 bootstrap replications to account for skewed methylation distributions

^d^Approximate p-value estimated from a Wald test of the normal-based bootstrapped estimate over its standard error. *P*-values > 0.1 are suppressed

^e^These seven assays were not pursued beyond the pilot phase and therefore have considerably fewer cancer and adjacent tissue samples analyzed

All estimates of mean percent methylation are adjusted for age, race/ethnicity, stage at diagnosis, tumor grade, and either adjusted for or stratified by ER/PR status

### DNA hypomethylation in cancer vs. adjacent and control mammoplasty samples

We found that the promoter regions of *TFF1* and *MAGEA1* were hypomethylated in cancer compared with adjacent samples (p < 10^-5^) and in cancer vs. control mammoplasty samples (p < 10^-4^; Tables 3 and 4). *MAGEA1* had high mean methylation levels in the control mammoplasty samples and adjacent samples (>80 % for both) but much lower DNA methylation levels in the cancer samples. *TFF1* also had high mean methylation levels in the control mammoplasty tissue (82 %), although methylation levels were lower in adjacent tissue (72 %), and lowest in cancer tissue (49 %). Cancer-associated hypomethylation of *TFF1* and *MAGEA1* was also observed by Illumina HumanMethylation450 analysis of DNA methylation in the TCGA database for breast cancer and paired normal samples (Fig. 2b, Panel b and Table 5). In addition, pyrosequencing revealed that the two studied DNA repeats, the tandem, juxtacentromeric satellite 2 (Sat2) and interspersed repeat LINE-1, displayed significant hypomethylation in cancer vs. adjacent samples (Table 3). However, the extent of hypomethylation for these highly repeated sequences was much less (5.4 and 1.6 %, respectively), which is not surprising given the very high copy number for these repeats.

**Table 5:** Methylation comparing cancer to paired adjacent samples, and correlation of methylation in invasive breast cancer samples with gene expression

	This study	TCGA database, within study region +/- 100 bp							TCGA database, within study region						
	Pyroseq^a^	Illum. 450 k: Mean % methylation					Assn. with expr.		Illum. 450 k: Mean % Methylation					Assn. with expr.	
Gene	# CpG	# CpG	Adjacent^b^ (N = 96)	Cancer^c^ (N = 96)	Diff^d^	*P*-value^e^	ρ^f^	*P*-value	# CpG	Adjacent^b^ (N = 96)	Cancer^c^ (N = 96)	Diff^d^	*P*-value^e^	ρ^f^	*P*-value
Promoter region															
BRCA1	11	13	4	4	0	NS	-0.19	0.0000	9	3	4	0	NS	-0.20	0.0000
CD44	8	5	4	9	5	0.0001	-0.18	0.0000	1	6	10	5	0.004	-0.18	0.0000
ESR1	5	2	67	40	-28	0.0000	-0.52	0.0000	2	67	40	-28	0.0000	-0.52	0.0000
GSTM2	8	3	4	16	13	0.0000	-0.13	0.0003	1	2	2	-1	0.0000	-0.13	0.0002
GSTP1	4	2	7	24	17	0.0000	-0.43	0.0000	0	---	---		---	---	---
MAGEA1	6	2	97	77	-21	0.0000	-0.23	0.0000	2	97	77	-20	0.0000	-0.23	0.0000
MSI1	5	2	3	6	3	0.0000	-0.20	0.0000	0	---	---		---	---	---
NFE2L3	14	1	39	48	9	0.0001	-0.55	0.0000	0	---	---		---	---	---
RASSF1	9	2	12	47	35	0.0000	-0.18	0.0000	1	9	49	41	0.0000	-0.16	0.0000
RUNX3	28	1	2	23	21	0.0000	-0.28	0.0000	0	---	---		---	---	---
SIX3	12	4	6	24	18	0.0000	-0.21	0.0000	2	4	26	22	0.0000	-0.20	0.0000
TFF1	5	4	77	46	-31	0.0000	-0.20	0.0000	1	74	47	-27	0.0000	-0.19	0.0000
Upstream of promoter															
EN1 ^g^	6	2	23	32	8	0.0000	0.12	0.001	0	---	---		---	---	---
PAX3	5	2	6	30	24	0.0000	-0.12	0.004	1	9	29	19	0.0000	-0.10	0.003
PITX2 ^g^	10	2	23	38	15	0.0000	0.22	0.0000	0	---	---		---	---	---
SGK1	6	4	9	21	12	0.0000	-0.10	0.003	2	5	17	12	0.0000	-0.11	0.003
Introns															
APC ^g^	4	11	6	28	22	0.0000	0.13	0.002	3	5	28	23	0.0000	0.12	0.004
EGFR	4	1	9	37	29	0.0000	-0.15	0.0000	1	9	37	29	0.0000	-0.15	0.0000
LHX2 ^g^	11	1	17	32	15	0.0000	0.24	0.0000	0	---	---		---	---	---
RFX1	4	0	---	---		---	---	---	0	---	---		---	---	---
SOX9	4	1	5	15	11	0.0000	-0.34	0.0000	0	---	---		---		

^a^Pyrosequencing (Pyroseq) assay coordinates are given in Table 1

^b^Non-cancer tissue adjacent to paired breast cancer from 96 patients in the TCGA Illumina Methylation450 database for genome-wide DNA methylation [14]

^c^Samples from the invasive component of the breast cancer from patients in the TCGA Methylation450 and expression (expr.; RNA-seq) databases [16, 17]

^d^Difference in mean % methylation for cancer minus that for the paired adjacent tissue for the 96 patients with both in the TCGA database

^e^From a dependent sample Wilcoxon Sign Rank test; P-values > 0.10 are suppressed

^f^Spearman correlation coefficient for methylation levels vs expression levels among all the invasive breast cancer samples in the TCGA database

^g^The four underlined genes were the only ones that had a positive association of cancer methylation in non-promoter regions with expression levels of the associated gene

### Cancer-associated aberrant methylation in adjacent tissue vs. control mammoplasty samples

A comparison that could be made with our pyrosequencing data, that is not available in the TCGA database for breast samples, is an analysis of cancer-adjacent tissue vs. breast tissue from cancer-free individuals. Comparing methylation levels of the adjacent samples in breast cancer patients and the control mammoplasty samples revealed that *RASSF1A* had the largest difference in mean methylation (Table 3). Only five other sequences displayed hypermethylation or hypomethylation in adjacent vs. control mammoplasty samples at the significance level of p < 0.01 (*SGK1*, LINE-1, *EGFR*, Sat2 and *TFF1*; Table 3) and only the first two of these at p ≤ 0.001. Surprisingly, the most statistically significant difference between methylation in adjacent tissue relative to control mammoplasty tissue was hypermethylation of LINE-1 (p < 10^-9^) as contrasted with the hypomethylation of this repeat in cancer vs. adjacent samples (p = 10^-4^). However, the magnitude of the expected [45] hypomethylation of LINE-1 repeats in cancer vs. adjacent tissue was small (-1.6 percentage points) and the magnitude of observed hypermethylation was modest (+4.1 percentage points). Mammoplasty control samples came from women who were younger (mean of 33 y, range 16-68) than the patients from whom the breast cancer samples originated (mean of 56 y, range 25-77), as would be expected given the availability of such samples. In addition, there are some differences in the cellular composition of breast tissue dependent upon whether it was derived from obese women, the likely source of most mammoplasty samples [46]. Therefore, the small differences in methylation, as seen for LINE-1, need to be interpreted with caution.

### Correlations between cancer-associated changes in DNA methylation and gene expression

An analysis of the Illumina HumanMethylation450 DNA methylation database for invasive breast cancers and the RNA-seq expression database for the same cancers in the TCGA collection [14] demonstrated that the methylation status of most of the studied regions was significantly associated with altered expression of the corresponding gene (Table 5). For this analysis, we focused on either the same small region studied by pyrosequencing in this study or that region extended by 100 bp on either side (Table 5). All the promoter regions for which we demonstrated cancer-linked hypermethylation by pyrosequencing (*BRCA1*, *CD44, GSTM2*, *GSTP1*, *MSI1, NFE2L3, RASSF1*, *RUNX3* and *SIX3*) exhibited an inverse correlation with expression among the cancers. Therefore, as expected [47], more promoter methylation was associated with lower expression levels. The two promoter regions displaying cancer hypomethylation (*TFF1* and *MAGEA1*) also displayed an inverse correlation between methylation among cancers and expression indicating that cancer-linked losses in promoter methylation were associated with increased (and abnormal) expression. Importantly, the only regions that displayed a positive correlation between methylation and expression among the breast cancers in the TCGA database were four far-upstream or intragenic regions for the genes *EN1*, *PITX2*, *APC* and *LHX2*.

### Insights into DNA hypermethylation positively associated with gene expression from the ENCODE database

We compared DNA methylation from ENCODE RRBS profiles of normal breast epithelial cells (HMEC) and several breast cancer cell lines, MCF-7 and T-47D (ENCODE/RRBS/HudsonAlpha Institute; [18, 19]). In addition, profiling of all mappable CpG sites in HMEC and the breast cancer cell line HCC1954 was available [15, 18]. As expected, differences in DNA methylation between promoter regions that we examined by pyrosequencing mostly mimicked the hypermethylation or hypomethylation observed in cancer vs. adjacent tissue or control mammoplasty tissue analyses by pyrosequencing (Additional file 1: Table S1).

Next we used ENCODE data to analyze transcriptome profiles available for HMEC, many other normal cell cultures and MCF-7 in the ENCODE database (ENCODE/RNA-seq/Cold Spring Harbor Lab) to elucidate the positive association shown in Table 5 between cancer DNA hypermethylation and gene expression for the pyrosequenced regions in *EN1*, *LHX2, PITX2* and *APC*. With respect to *EN1,* methylation of its far-upstream region was positively associated with expression in a comparison of normal cell cultures. Normal myoblast and myotube cultures, which strongly and preferentially express *EN1* were significantly hypermethylated in this far-upstream region when compared with other studied cell cultures and tissues [48] including HMEC and MCF-7 cells (Fig. 1c and d). Unlike myoblasts and myotubes, the MCF-7 breast cancer cell line was hypermethylated not only in this region but throughout the body of the *EN1* gene, which may explain why MCF-7 cells did not express *EN1* while myoblasts and myotubes did. Similar to the studied *EN1* far-upstream region, intron 3 of *LHX2* and intron 1 of *PITX2*, exhibited muscle lineage hypermethylation directly associated with highly specific expression in myoblasts (data not shown, [18]). In contrast, *APC* is broadly expressed among diverse cell types.

### Histone modifications and gene expression from ENCODE

We also examined the pyrosequenced regions in ENCODE histone modification profiles, which were available for HMEC but not for MCF-7 (ENCODE/Histone Modifications by ChIP-seq/Broad Institute). As expected, promoter hypermethylation in cancer was usually in regions displaying active promoter-type histone modifications in HMEC (Additional file 1: Table S1). These histone modification profiles distinguish between chromatin regions that are predicted to be active promoters (histone H3 lysine-4 trimethylation, H3K4me3, and H3K27 acetylation, H3K27ac), silenced regions (H3K27me3), active enhancers (H3K4me1 and H3K27ac), and poised promoters or enhancers (H3K4 methylation sometimes with H3K27me3 but without H3K27ac)[49]. The histone methylation profiles (Additional file 1: Table S2) also indicate that two of the studied regions far downstream of *EGFR* (1.35 kb downstream of the TSS in intron 1) and *SOX9* (2 kb downstream of TSS, in intron 2) have the chromatin modifications typical of active promoters in HMEC cultures, in which these genes are expressed.

Histone modification profiles for HMEC cultures were also very informative for the four intragenic or far-upstream regions that displayed breast cancer-associated DNA hypermethylation as well as a positive association between DNA methylation and expression among TCGA breast cancers (Additional file 1: Table S2). In HMEC cultures, the examined *EN1, PITX2* and *LHX2* chromatin regions all exhibited enrichment in H3K27me3. This histone mark is often, but not always, associated with repression and frequently found in DNA regions in normal cells (especially stem cells) that become hypermethylated during carcinogenesis [50]. The pyrosequenced region in *APC*, unlike the above three gene regions, exhibited the histone marks of an active promoter (H3K4me3 and H3K27ac) in HMEC. However, although this region is 30 kb downstream of the *APC* TSS defined by the isoform NM_001127511, it overlaps an alternative promoter associated with isoform NM_000038. Both isoforms encode the APC protein, although their promoters are separated by 30 kb, and both are functionally important [51]. HMEC cultures express both isoforms abundantly, as indicated by histone modification and RNA profiling in ENCODE databases (Additional file 1: Table S2). However, TCGA methylation profiles showed that only the downstream alternative promoter region becomes hypermethylated in breast cancers. The average percent methylation at the upstream and downstream promoters in invasive breast cancer in the TCGA database were 10 and 28, respectively, while those for paired normal tissue were 11 and 6.

## Discussion

Using a candidate gene approach on a large, ethnically diverse set of subjects, we compared not only invasive breast cancer and adjacent histologically normal tissue (as in the TCGA Illumina HumanMethylation450 database [14]), but also control samples of reductive mammoplasty tissue from non-cancer patients using a quantitative, gold-standard method for DNA methylation analysis (bisulfite/pyrosequencing) amenable to archival FFPE samples. Our pyrosequencing analysis of DNA methylation involved promoter DNA regions, regions far upstream of genes, intragenic regions and high-copy interspersed or tandem DNA repeats. In addition, DNA methylation, transcriptome and histone modification profiles from TCGA or ENCODE whole-genome databases were used to enhance the analysis. A limitation of our study of aberrant DNA methylation in breast cancer is that clinical samples such as ours include cell types other than breast epithelial cells. Therefore, the methylation levels estimated in our study represent an average across many cell types. Nonetheless, the similarities between hyper- or hypomethylation determined in our bioinformatic comparisons of DNA methylation in cancer-derived and normal mammary epithelial cell cultures (Additional file 1: Tables S1 and S2) and aberrant DNA methylation from our pyrosequencing study of maligant and non-cancerous breast tissues (Table 3) argue for our analysis indicating DNA changes, at least in part, in the epithelial cell populations in cancers vs. non-cancerous breast samples.

Besides confirming that a wide variety of DNA sequences display hyper- or hypomethylation in a large, diverse collection of invasive breast cancers vs. adjacent tissue, we demonstrated significant hyper- or hypomethylation in six of the 16 DNA regions examined in both 15 - 18 reduction mammoplasty samples and more than 100 histologically normal tissue samples adjacent to the breast cancers. These six DNA sequences were in promoter regions (*RASSF1* and *TFF1*), an intron (*EGFR*), a far-upstream (*SGK1*) gene regions or DNA repeats (LINE-1 and Sat2). If control mammoplasty samples are mimicking the epigenetics of normal breast tissue, then our results suggest a field effect that could include changes which predispose to carcinogenesis [12]. The adjacent tissue samples used in our comparison had been carefully evaluated morphologically and histologically for no evidence of malignancy. In addition, the lack of evidence for a field effect for most of the studied DNA regions, including for regions with frequent hypermethylation in the cancer tissue (e.g., *RFX1* and *EN1*), is consistent with a field effect rather than contamination of adjacent samples with tumor tissue.

Field effects for DNA methylation changes in *RASSF1, EGFR* and *TFF1* might be important in influencing pre-neoplastic changes in gene expression relevant to tumor development. *RASSF1* is a tumor suppressor gene that regulates apoptotic and cell cycle checkpoints [52]. *RASSF1* hypermethylation has been detected in carcinoma in situ and invasive breast cancer and is inversely correlated with RNA and protein expression levels [24, 53, 54] and overall survival [55]. Like overexpression of HER-2 protein, overexpression of EGFR protein, another member of the epidermal growth factor/tyrosine kinase family, is related to multiple drug resistance and decreased patient survival [56, 57]. We demonstrated significant hypermethylation of *EGFR* at part of the extended promoter-like chromatin region (see below) in intron 1 by comparing cancers with adjacent tissue. Hypermethylation in this region was also seen in the comparison of histologically normal, cancer-adjacent tissue and control mammoplasty tissue. Given the proto-oncogene status assigned to *EGFR*, it is not yet clear what role hypermethylation of *EGFR* might play in breast cancer progression.

Expression of *TFF1,* a gene encoding a small secretory peptide implicated in preserving mucosa in the intestinal track, is associated with promoter hypomethylation in cultured cells and in breast cancers [29, 58]. We found hypomethylation of the promoter of this estrogen-inducible gene in both cancer vs. adjacent tissue and in adjacent vs. control mammoplasty tissue. Expression of *TFF1* in breast cancer may be associated with a poor outcome based upon breast cancer cell lines and a mouse model [59]. However, a recent study of breast cancer patients found that *TFF1* expression was greater for ER/PR positive breast cancers, which generally have a better prognosis than ER/PR negative breast cancers [60]. Similarly, we found that ER/PR positive tumors showed greater hypomethylation compared with ER/PR negative tumors.

One surprising result from our analysis was that the extended promoter region of *BRCA1* did not show significant hypermethylation in breast cancer relative to adjacent tissue or control mammoplasty tissue despite the fact that we chose a promoter region for analysis similar to or overlapping those employed in other studies, many of which did find *BRCA1* hypermethylation in breast cancer [28, 31, 61–64]. The first three of these studies used end-point methylation-specific PCR, which is extremely sensitive for detection of any DNA methylation but is not quantitative, and these studies reported only the percentages of samples that were called as methylated. We found very low average levels of methylation in the *BRCA1* promoter region in all samples (1.4, 1.6 and 3 % for control mammoplasty, adjacent and cancer samples, respectively) including a few outliers with considerable methylation. In a study using MALDI-TOF mass array analysis of 48 FFPE samples, only five of the 17 tested CpG sites displayed hypermethylation in breast cancer vs. matching control tissue, and the extent of hypermethylation at these five sites was surprising high (averages of about 90 % for cancers vs. about 10 % for controls) [63]. However, as in our study, MALDI-TOF [65] and methylation-specific multiplex ligation assays [64] by two other groups, each using flash-frozen breast cancers and matching control tissue, revealed only low percentages of cancers with greater *BRCA1* methylation compared with controls. Moreover, in their studies and ours there was much more frequent cancer hypermethylation at many other tested promoter regions. Similarly, bioinformatic analysis of methylation levels in the HumanMethylation450 TCGA database revealed insignificant differences between breast cancer and adjacent tissue for the *BRCA1* promoter region that we examined (Table 5).

Our results from pyrosequencing that intronic sequences far downstream of the canonical promoter region (*EGFR*, *LHX2*, *SOX9* and *RFX1*) and intergenic sequences upstream of the promoter (*PAX3* and *EN1*) were significantly hypermethylated in breast cancer may be related to new understandings of the transcription-regulatory roles played by DNA methylation in intragenic and distant intergenic regions [2, 66]. For example, sequences considerably downstream of the TSS may be part of the functional promoter or of transcription-elongation regulatory elements such that local methylation could alter gene expression levels. This may be the case for the pyrosequenced regions of *EGFR* (1.35 kb downstream of the TSS in intron 1) and *SOX9* (2 kb downstream of TSS, in intron 2) far downstream of the TSS. These two regions displayed hypermethylation in cancer. In normal HMEC, where these two genes are actively transcribed, ENCODE histone modification profiling (Additional file 1: Table S2) indicates that the studied regions overlap large chromatin segments with histone modifications typical of active promoters starting in the canonical promoter region and continuing into the 5’ intragenic region [13].

The importance of not restricting analysis of cancer-linked aberrant DNA methylation to standard promoter regions is also apparent from recent studies providing evidence that DNA hypermethylation is implicated in alternative promoter usage; regulating splicing of RNA; and, in certain intragenic regions, in upregulating expression [2, 66]. Indeed, examples of the latter were seen in our bioinformatic analyses of databases for DNA methylation and expression in breast cancers (TCGA) and in cultured cells (ENCODE). For example, we found that DNA hypermethylation at *APC* was positively associated with increased expression among breast cancers (TCGA database). While the examined *APC* region is 30 kb downstream of the TSS in intron 1 of one *APC* isoform expressed in HMEC, it is also in the promoter region of another protein-coding isoform of the gene transcribed in HMEC. Therefore, the cancer-associated hypermethylation of this *APC* intron/promoter region might help regulate levels of alternate promoter usage for this gene.

The other three breast cancer-associated DNA hypermethylated regions which displayed significantly more expression in breast cancers with higher levels of DNA methylation are 5.6 kb upstream of *EN1*, 7 kb upstream of *PITX2* or 4 kb downstream of the TSS of *LHX2*. These genes code for homeobox-containing transcription factors important in development. The location of these regions and their association with normally repressive H3K27me3 in HMEC cultures (Additional file 1: Table S2) suggest that the positive correlation of DNA methylation at these regions with transcription may be due to their playing a role in controlling the borders of active promoter regions and counteracting the spread of H3K27me3-repressive chromatin into the core promoter [67].

## Conclusions

We identified frequent DNA methylation changes in invasive breast cancer at a variety of genome locations and found evidence for an extensive field effect in breast cancer. Empirical and bioinformatic analyses of these gene regions provide further examples of the power of combining a candidate gene approach and bioinformatics using publicly available databases to better understand the importance of cancer epigenetic changes.

## Additional file

Additional file 1:
**Table S1.** “Promoter test region genes” and “**Table S2.** Non-promoter test region genes”. Two tables containing sources of and interpretation of DNA methylation, histone modification and expression data for the selected genetic regions in the pyrosequencing study. **Table S1.** describes regions found in gene promoter locations. **Table S2.** describes regions found in intragenic or far-upstream regions to genes. (DOCX 54 kb)

## Abbreviations

TCGA: The Cancer Genome Atlas
ENCODE: Encyclopedia of DNA Elements
FFPE: Formalin-fixed, paraffin-embedded
RLUs: Relative light units
S/N: Signal to noise
RRBS: Reduced representation bisulfite sequencing adjacent tissue, histologically normal tissue adjacent to the cancer
HMEC: Human mammary epithelial cells
5mC: 5-Methylcytosine
ER: Estrogen receptor
PR: Progesterone receptor
HER2: Human epidermal growth factor receptor-2
SD: Standard deviation
TSS: Transcription start site
