# Supplementary material for: Exploring DNA methylation changes in promoter, intragenic, and intergenic regions as early and late events in breast cancer formation
Source: BMC Cancer. 2015 Oct 29;15:816. doi: 10.1186/s12885-015-1777-9 (PMC4625569; doi:10.1186/s12885-015-1777-9)
Supplement: Additional file 1: — Table S1. “Promoter test region genes” and “Table S2. Non-promoter test region genes”. Two tables containing sources of and interpretation of DNA methylation, histone modification and expression data for the selected genetic regions in the pyrosequencing study. Table S1. describes regions found in gene promoter locations. Table S2. describes regions found in intragenic or far-upstream regions to genes. (DOCX 54 kb) [file 12885_2015_1777_MOESM1_ESM.docx]

| **Table S1. Promoter test regions for bisulfite pyrosequencing** | | | | | | | | |
| --- | --- | --- | --- | --- | --- | --- | --- | --- |
| **Gene/RNA isoform^1^** | **Test region coordinates (hg19)** | **Distance from TSS** | **Hyper- or hypometh. in a breast cancer cell line from RRBS database (ENCODE/RRBS/HudsonAlpha)^2^** | **Meth. in a breast cancer cell line & HMEC from bisulfite-seq (ENCODE/DNA Methylation/USC)[**[**1**](#_ENREF_1)**]^2^** | **Signal for H3K4 or H3K27 modification for HMEC (ENCODE/Histone Modifications by ChIP-seq/Broad Institute)^2^** | **Hypermeth. in breast cancer vs. cancer-adj./non-cancerous mammoplasty (this study)^3^** | **Hypometh. in breast cancer vs. cancer-adj./non-cancerous mammoplasty (this study)^3^** | **Possible role of sequence in regulation of gene expression in normal cell cultures from ENCODE data^2^ or indicated references** |
| BRCA1/NM_007294 | chr17: 41277463-41277365 | +37 to +135 | No RRBS data | HMEC, HCC1954, & many normal tissue types unmeth. | H3K4me3 & H3K27ac | near signif./no | no/no | Promoter hypermeth. reported to be assoc. with gene repression [[2](#_ENREF_2)] |
| CD44/ NM_001202557 | chr11:35160374-35160443 | -43 to +26 | No RRBS data | HMEC & HCC1954 unmeth. | H3K4me3 & H3K27ac | no/no | no/no | Part of promoter active in many cell types; promoter hypermeth. is implicated in gene repression [[3](#_ENREF_3)] |
| ESR1/NM_001122740 | chr6:152129110-152129167 | +656 to +713 | No RRBS data | HMEC & HCC1954 unmeth. | H3K4me3, H3K27ac, & H3K27me3 | no/no | no/no | No expression in HMEC. MCF-7 shows txn; this region could be an alternate promoter; previously reported that MCF-7 expresses *ESR1* & is meth. in this region [[4](#_ENREF_4)]; promoter meth. is inversely assoc. with expression in breast cancers [[5](#_ENREF_5), [6](#_ENREF_6)] |
| GSTM2 /NM_000848 | chr1: 110210582-110210641 | -62 to -3 | No RRBS data | HMEC unmeth. & HCC1954 highly meth. | Weak H3K4me3 & H3K27ac | yes/yes | no/no | Part of a promoter region; promotor hypermeth. is assoc. with gene repression [[7](#_ENREF_7)] |
| GSTP1/NM_000852 | chr11: 67351205-67351215 | +139 to +149 | Sparse RRBS data | HMEC unmeth. & HCC1954 highly meth. | H3K4me3 & H3K27ac | yes/no | no/no | Part of the immediate downstream promoter region; promoter hypermeth. is assoc. with gene repression [[8](#_ENREF_8)] |
| MAGEA1/NM_004988 | chrX:152486180-152486129 | -13 to -64 | No RRBS data | HMEC highly meth. & HCC1954 unmeth. | Little or none | no/no | yes/yes | Promoter hypermeth. represses txn. in non-germline cells [[9](#_ENREF_9)] |
| MSI1/NM_002442 | chr12:120807571-120807474 | -588 to -491 | No RRBS data | HMEC unmeth. & HCC1954 highly meth. | H3K27ac & H3K27me3 | yes/yes | no/no | Might be part of an extended upstream regulatory region |
| NFE2L3/NM_004289 | chr7:26192663-26192744 | +816 to +897 | MCF-7 & T-47D (& many other cancer cell lines) hypermeth. vs. HMEC & many normal tissues, including breast) but MCF-7 is partly meth. | HMEC partially meth & HCC1954 more highly meth. | H3K4me3 | no/no | no/no | Part of an extended TSS-downstream promoter region |
| RASSF1/NM_170713 | chr3: 50378294-50378232 | +74 to +134 | HMEC partly meth.; MCF-7 & T-47D (& many other cancer cell lines) hypermeth. vs. HMEC & many normal tissues, including breast) | HMEC unmeth. & HCC1954 highly meth. | H3K4me1 & H3K4me3 | yes/yes | no/no | Part of the immediate TSS-downstream promoter region at which DNA meth. is implicated in silencing txn [[10-12](#_ENREF_10)] |
| RUNX3/NM_004350 | chr1:25256198-25256306 | +464 to +572 | MCF-7 & T-47D (& many other cancer cell lines) partly or highly hypermeth. vs. HMEC & many normal tissues, including breast | HMEC unmeth. & HCC1954 partial meth. | H3K4me3 & H3K27me3 | yes/near signif. | no/no | Part of an extended TSS-downstream promoter region; promoter hypermeth. is assoc. with downregulation of txn. [[13](#_ENREF_13)] |
| SIX3/NM_005413 | chr2:45169609-45169529 | +492 to +572 | MCF-7 (& many other cancer cell lines, but not T-47D) hypermeth. vs. HMEC & many normal tissues, including breast) | HMEC unmeth. & HCC1954 highly meth. | Weak H3K4me3 & H3K27me3 | yes/near signif | no/no | Part of an extended TSS-downstream promoter region; gene not expr. in HMEC or MCF-7 nor most normal cell cultures in ENCODE RNA-seq database |
| TFF1/NM_003225 | chr21:43786664-43786628 | -20 to +16 | Hypometh. in MCF-7 vs. HMEC & most normal tissues | High meth. in both HMEC & HCC1954 | Little or none | no/no | yes/yes | Part of promoter; promoter hypometh.assoc. with gene expr. [[14](#_ENREF_14)] [[15](#_ENREF_15)] |
| ^1^ Where there are multiple RefSeq RNA isoforms and expression in HMEC cells by RNA-seq (ENCODE/Cold Spring Harbor), the RNA isoform closest to the predominant HMEC RNA was used to determine the TSS. The assays were designed for the coding strand. RRBS, reduced representation bisulfite sequencing for whole-genome methylation profiling; assoc., association, meth., methylated or methylation; unmeth, unmethylated; adj., non-cancerous tissue adjacent to cancer; txn, transcription; expr., expressed; signif., significant | | | | | | | | |
| ^2^RRBS, bisulfite-seq [[1](#_ENREF_1)], histone modification and transcription databases are from <http://genome.ucsc.edu> [[16](#_ENREF_16)] ; H3K4me3, H3 lysine-4 trimethylation; H3 K4me1, H3 lysine-4 monomethylation; H3K4me2, H3 lysine-4 dimethylation; H3K27ac, H3 lysine-27 acetylation; H3K27me3, H3 lysine-27 trimethylation; associations of these histone modifications with active promoters, enhancers, or silenced cis-acting transcription regulatory elements is explained in Results and in Reference 16. | | | | | | | | |
| ^3^Significant hyper- or hypomethylation of breast cancer vs. histologically normal cancer-adjacent tissue; after the slash is shown the comparison for breast cancer vs. non-cancerous reduction mammoplasty samples; determination of methylation by pyrosequencing (p<0.01 but usually much lower; see Tables 3 in the main article) | | | | | | | | |

| **Table S2. Non-promoter test regions for bisulfite pyrosequencing ^1^** | | | | | | | | |
| --- | --- | --- | --- | --- | --- | --- | --- | --- |
| **Gene/RNA isoform1** | **Test region coordinates (hg19)** | **Distance from TSS** | **Hyper- or hypometh. in a breast cancer cell line from RRBS database (ENCODE/RRBS/HudsonAlpha)** | **Meth. in a breast cancer cell line & HMEC from bisulfite-seq (ENCODE/DNA Methylation/USC)** | **Signal for H3K4 or H3K27 modification for HMEC (ENCODE/Histone Modifications by ChIP-seq/Broad Institute)** | **Hypermeth. in breast cancer vs. cancer-adj./non-cancerous (this study)** | **Hypometh. in breast cancer vs. cancer-adj./non-cancerous (this study)** | **Possible role of sequence in regulation of gene expression in normal cell cultures from ENCODE data^2^ or indicated references** |
| APC/NM_001127511 or NM_000038 | chr5: 112073426-112073445 | +30224 to +30243 or -130 to -111 (alternate intron or promoter, depending on gene isoform) | No RRBS data | HMEC unmeth. & HCC1954 highly meth. | H3K4me3 & H3K27ac | yes/near-signif. | no/no | Promoter hypermeth. is assoc. with gene repression [[17](#_ENREF_17)] & ENCODE profiles for H3K4me3 and H3K27ac as well as profiles for 5’ cap analysis gene expression (ENCODE/CAGE/Riken Omics) tags indicate that two promoter regions about 30 kb apart are both used for expression in HMEC & the downstream one overlaps the analyzed site in the present study |
| EGFR/NM_201283 | chr7: 55088080-55088104 | +1355 to +1379 | MCF-7 & T-47D (& many other cancer cell lines) hypermeth. vs. HMEC & many normal tissues including breast | HMEC & HCC1954 unmeth. | H3K4me3 & H3K27ac | yes/yes | no/no | Part of an extended downstream promoter region; MCF-7 hypermeth. & decreased txn vs. HMEC correlates with MCF-7 promoter hypermeth.; promoter hypermeth. is assoc. with down regulation of gene expr. [[18](#_ENREF_18)] |
| EN1/NM_001426 | chr2:119611385-119611338 | -5579 to -5626 | MCF-7 & T-47D (& many other cancer cell lines) hypermeth. vs. HMEC & many normal tissues including breast | HMEC unmeth. & HCC1954 highly meth. | Weak H3K4me3, H3K27ac, & H3K27me3 | yes/yes | no/no | Part of a far-upstream repressive or down-modulatory element; both MCF-7 and HMEC do not express this gene |
| LHX2/NM_004789 | chr9:126777854-126777983 | +3966 to +4095 | MCF-7 & T-47D (& many other cancer cell lines) hypermeth. vs. HMEC & many normal tissues including breast | HMEC unmeth. & HCC1954 highly meth. | H3K27me3 | yes/yes | no/no | Central part (intron 3) of gene body; this gene is silent in most tested cell cultures; it is much more highly expressed in MCF-7 (and HeLa) than in HMEC (& most nonmalignant cell cultures with ENCODE RNA-seq data) & is hypermeth. in the gene body, but not the promoter, in MCF-7 (& HeLa) |
| PAX3 / CCDC140 /NM_001127366 | chr2:223170608-223170643 | -6928 to -6893 | MCF-7 & T-47D hypermeth. vs. HMEC & many normal tissues including breast | HMEC unmeth. & HCC1954 highly meth. | H3K4me2, H3K27me3 | yes/yes | no/no | DNA hypermeth. in Mb & melanocytes positively assoc. with gene expr.; meth. might down modulate txn. but not suppress it or counteract a silencer [[19](#_ENREF_19)] |
| PITX2/NM_000325 or NM_153426 | chr4:111562566-111562677 | -18312 to -18413 or +602 - +713 | MCF-7 & T-47D (& many other cancer cell lines) hypermeth. vs. HMEC & many normal tissues including breast | HMEC unmeth. & HCC1954 highly meth. | H3K27me3 | yes/near signif. | no/no | Part of an extended TSS-downstream promoter region |
| RFX1/NM_002918 | chr19: 14089984-14089969 | +27150 to +27165 | HMEC partly meth, MCF-7 & T-47D highly meth. relative to breast tissue & majority of normal tissues | HMEC partially meth & HCC1954 more highly meth. | Little or none | yes/yes | no/no | Expr. in HMEC & MCF7. DNA hypometh. in LCL and ESC assoc. with predicted enhancer activity in lung fibroblasts, lymphoblasts, and ESC from histone modifications; transfection assays in a glioma cell line demonstrated that enhancer activity & DNA hypermethylation in glioma cell lines & tissues is assoc. with less gene expr. [[20](#_ENREF_20)] |
| SGK1/NM_005627 & NM_001143678 | chr6: 134638893-134638831 | -14823 to -14761 or +303 to +365 | No RRBS data at this sequence but RRBS data within nearby (<100 bp away) indicated hypermeth. in MCF-7 (but not T47D) vs. HMEC & many normal tissues including breast | HMEC unmeth. & HCC1954 highly meth. | H3K4me3 | yes/yes | no/no | Part of an alternative, far upstream extended promoter for an SGK1 isoform, which is among the isoforms expressed in HMEC |
| SOX9/NM_000346 | chr17: 70119151-70119195 | +1990 to +2034 | MCF-7 (& many other cancer cell lines, but not T-47D) hypermeth. vs. HMEC & many normal tissues including breast) | HMEC unmeth. & HCC1954 highly meth. | H3K4me3 & H3K27ac | yes/near signif. | no/no | Uncertain or possibly no function of cancer-assoc. hypermeth. in this gene body (intron 2) region because no simple correlations between meth. & gene expr. |
| ^1^ Abbreviations and notation are explained in Table S1 with the addition of CAGE, 5’ cap analysis of gene expression (ENCODE/CAGE/RIKEN Omics Science Center), an ENCODE database available at <http://genome.ucsc.edu> | | | | | | | | |

**Supplemental References**

1. Hon GC, Hawkins RD, Caballero OL, Lo C, Lister R, Pelizzola M, Valsesia A, Ye Z, Kuan S, Edsall LE *et al*: **Global DNA hypomethylation coupled to repressive chromatin domain formation and gene silencing in breast cancer**. *Genome research* 2012, **22**(2):246-258.

2. Alkam Y, Mitomi H, Nakai K, Himuro T, Saito T, Takahashi M, Arakawa A, Yao T, Saito M: **Protein expression and methylation of DNA repair genes hMLH1, hMSH2, MGMT and BRCA1 and their correlation with clinicopathological parameters and prognosis in basal-like breast cancer**. *Histopathology* 2013, **63**(5):713-725.

3. Yan P, Mühlethaler A, Bourloud KB, Beck MN, Gross N: **Hypermethylation-mediated regulation of CD44 gene expression in human neuroblastoma**. *Genes Chromosomes Cancer* 2003, **36**(2):129-138.

4. Rasti M, Arabsolghar R, Khatooni Z, Mostafavi-Pour Z: **p53 Binds to estrogen receptor 1 promoter in human breast cancer cells**. *Pathol Oncol Res* 2012, **18**(2):169-175.

5. Martínez-Galán J, Torres-Torres B, Núñez MI, López-Peñalver J, Del Moral R, Ruiz De Almodóvar JM, Menjón S, Concha A, Chamorro C, Ríos S *et al*: **ESR1 gene promoter region methylation in free circulating DNA and its correlation with estrogen receptor protein expression in tumor tissue in breast cancer patients**. *BMC Cancer* 2014, **14**:59.

6. Shenker NS, Flower KJ, Wilhelm-Benartzi CS, Dai W, Bell E, Gore E, El Bahrawy M, Weaver G, Brown R, Flanagan JM: **Transcriptional implications of intragenic DNA methylation in the oestrogen receptor alpha gene in breast cancer cells and tissues**. *BMC Cancer* 2015, **15**(1):337.

7. Peng DF, Razvi M, Chen H, Washington K, Roessner A, Schneider-Stock R, El-Rifai W: **DNA hypermethylation regulates the expression of members of the Mu-class glutathione S-transferases and glutathione peroxidases in Barrett's adenocarcinoma**. *Gut* 2009, **58**(1):5-15.

8. Zhang W, Jiao H, Zhang X, Zhao R, Wang F, He W, Zong H, Fan Q, Wang L: **Correlation between the expression of DNMT1, and GSTP1 and APC, and the methylation status of GSTP1 and APC in association with their clinical significance in prostate cancer**. *Mol Med Rep* 2015, **12**(1):141-146.

9. Cannuyer J, Loriot A, Parvizi GK, De Smet C: **Epigenetic hierarchy within the MAGEA1 cancer-germline gene: promoter DNA methylation dictates local histone modifications**. *PloS one* 2013, **8**(3):e58743.

10. Pasquali L, Bedeir A, Ringquist S, Styche A, Bhargava R, Trucco G: **Quantification of CpG island methylation in progressive breast lesions from normal to invasive carcinoma**. *Cancer Lett* 2007, **257**(1):136-144.

11. Dammann R, Yang G, Pfeifer GP: **Hypermethylation of the cpG island of Ras association domain family 1A (RASSF1A), a putative tumor suppressor gene from the 3p21.3 locus, occurs in a large percentage of human breast cancers**. *Cancer Res* 2001, **61**(7):3105-3109.

12. Xu J, Shetty PB, Feng W, Chenault C, Bast RC, Issa JP, Hilsenbeck SG, Yu Y: **Methylation of HIN-1, RASSF1A, RIL and CDH13 in breast cancer is associated with clinical characteristics, but only RASSF1A methylation is associated with outcome**. *BMC Cancer* 2012, **12**:243.

13. Subramaniam MM, Chan JY, Soong R, Ito K, Ito Y, Yeoh KG, Salto-Tellez M, Putti TC: **RUNX3 inactivation by frequent promoter hypermethylation and protein mislocalization constitute an early event in breast cancer progression**. *Breast Cancer Res Treat* 2009, **113**(1):113-121.

14. Martin V, Ribieras S, Song-Wang XG, Lasne Y, Frappart L, Rio MC, Dante R: **Involvement of DNA methylation in the control of the expression of an estrogen-induced breast-cancer-associated protein (pS2) in human breast cancers**. *J Cell Biochem* 1997, **65**(1):95-106.

15. Buache E, Etique N, Alpy F, Stoll I, Muckensturm M, Reina-San-Martin B, Chenard MP, Tomasetto C, Rio MC: **Deficiency in trefoil factor 1 (TFF1) increases tumorigenicity of human breast cancer cells and mammary tumor development in TFF1-knockout mice**. *Oncogene* 2011, **30**(29):3261-3273.

16. Myers RM, Stamatoyannopoulos J, Snyder M, Dunham I, Hardison RC, Bernstein BE, Gingeras TR, Kent WJ, Birney E, Wold B *et al*: **A user's guide to the encyclopedia of DNA elements (ENCODE)**. *PLoS Biol* 2011, **9**(4):e1001046.

17. Zmetakova I, Danihel L, Smolkova B, Mego M, Kajabova V, Krivulcik T, Rusnak I, Rychly B, Danis D, Repiska V *et al*: **Evaluation of protein expression and DNA methylation profiles detected by pyrosequencing in invasive breast cancer**. *Neoplasma* 2013, **60**(6):635-646.

18. Montero AJ, Díaz-Montero CM, Mao L, Youssef EM, Estecio M, Shen L, Issa JP: **Epigenetic inactivation of EGFR by CpG island hypermethylation in cancer**. *Cancer Biol Ther* 2006, **5**(11):1494-1501.

19. Tsumagari K, Baribault C, Terragni J, Varley KE, Gertz J, Pradhan S, Badoo M, Crain CM, Song L, Crawford GE *et al*: **Early de novo DNA methylation and prolonged demethylation in the muscle lineage**. *Epigenetics* 2013, **8**(3):317-332.

20. Ohashi Y, Ueda M, Kawase T, Kawakami Y, Toda M: **Identification of an epigenetically silenced gene, RFX1, in human glioma cells using restriction landmark genomic scanning**. *Oncogene* 2004, **23**(47):7772-7779.
